# Pure Androgen-Secreting Adrenal Adenoma Associated with Resistant Hypertension

**DOI:** 10.1155/2013/356086

**Published:** 2013-05-29

**Authors:** René Rodríguez-Gutiérrez, Mario Arturo Bautista-Medina, Ana Eugenia Teniente-Sanchez, Maria Azucena Zapata-Rivera, Juan Montes-Villarreal

**Affiliations:** Endocrinology Division, University Hospital “Dr. José E. González,” Universidad Autonoma de Nuevo León, 64460 Monterrey, NL, Mexico

## Abstract

Pure androgen-secreting adrenal adenoma is very rare, and its diagnosis remains a clinical challenge. Its association with resistant hypertension is uncommon and not well understood. We present an 18-year-old female with a 10-year history of hirsutism that was accidentally diagnosed with an adrenal mass during the evaluation of a hypertensive crisis. She had a long-standing history of hirsutism, clitorimegaly, deepening of the voice, and primary amenorrhea. She was phenotypically and socially a male. FSH, LH, prolactin, estradiol, 17-hydroxyprogesterone, and progesterone were normal. Total testosterone and DHEA-S were elevated. Cushing syndrome, primary aldosteronism, pheochromocytoma, and nonclassic congenital adrenal hyperplasia were ruled out. She underwent adrenalectomy and pathology reported an adenoma. At 2-month followup, hirsutism and virilizing symptoms clearly improved and blood pressure normalized without antihypertensive medications, current literature of this unusual illness and it association with hypertension is presented and discussed.

## 1. Introduction

Benign and malignant tumors of the adrenal gland might be functional or silent. The majority of these tumors are benign, nonfunctioning adenomas that are incidentally discovered on abdominal image studies. Others are functional adenomas able to secrete cortisol, aldosterone, or less commonly androgens or estrogens [1]. Pure androgen-secreting adrenal tumors are very unusual, and their diagnosis represents a clinical challenge. Hirsutism and virilization syndrome, characterized by clitorimegaly, male pattern baldness, and deepening of the voice along with menstrual irregularities are the most common findings [2–4]. Resistant hypertension is also a clinical feature in these cases, and it is defined as arterial hypertension above goals in spite of the concurrent use of three different antihypertensive drugs of different classes, including a diuretic [5].

The association of pure androgen-secreting adrenal tumors with hypertension has exceptionally been reported. Most of the cases have been carcinomas and mixed hormone-secreting tumors [6–8]. Even though it is well known that testosterone can increase blood pressure and epidemiological studies have demonstrated a higher blood pressure in males than females, most studies agree that androgens are only an aggravating factor rather than the exclusive cause of resistant hypertension [9, 10]. 

Herein, we present the case of an 18-year-old female with a 10-year history of hirsutism, virilization, and primary amenorrhea associated to an incidental adrenal mass found after a hypertensive crisis. 

## 2. Case Report

**Figure 1:**
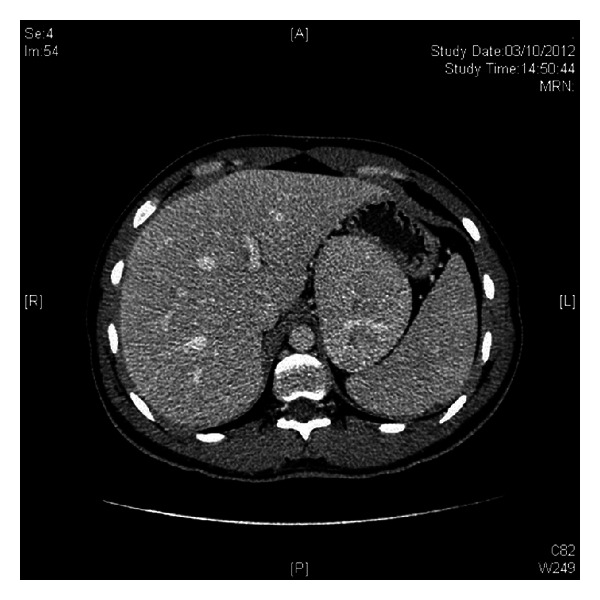


**Figure 2:**
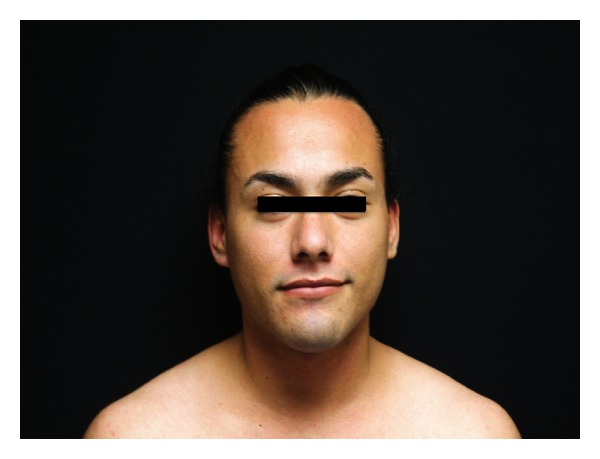


**Figure 3:**
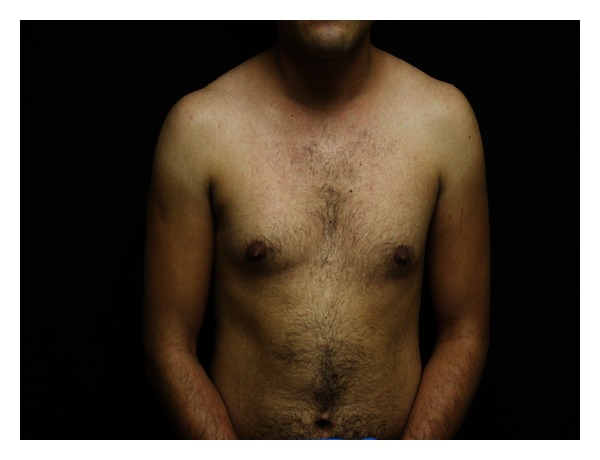


**Figure 4:**
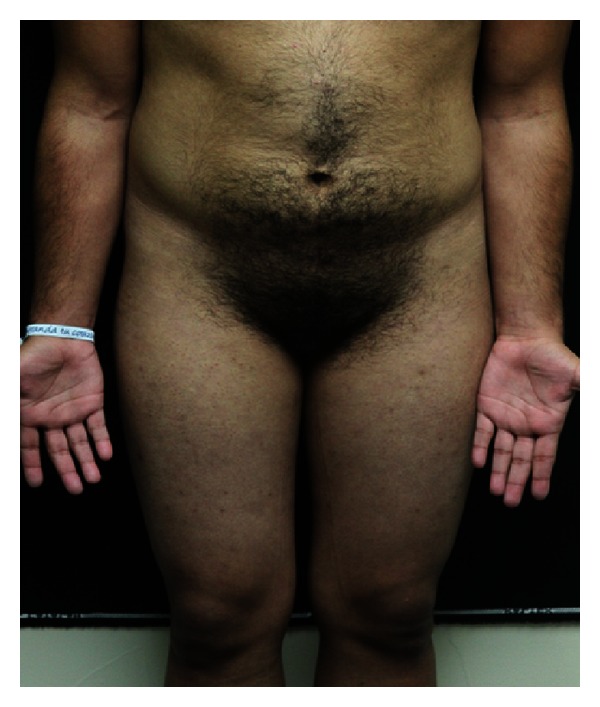


An 18-year-old female with past medical history of hirsutisim and hypertension was referred to the endocrinology clinic for assessment of an adrenal mass and resistant hypertension. She had a normal childbirth, no medical illness, and a normal sexual, social, and physiological development during her infancy and childhood. At age eight, she began to notice progressive hirsutism. However, she never asked for medical advice. Six years later, she was diagnosed with hypertension that was uncontrolled with full-dose telmisartan, metoprolol, prazosin, and nifedipine. She never had menarche. Two months before our evaluation, she presented a hypertensive crisis with right-sided hemiplegia and severe chest pain that relapsed after blood pressure was controlled. MRI was negative for stroke and electrocardiogram, and heart enzymes were normal. During the evaluation, a chest-abdomen CT scan was performed and a left adrenal mass of 10 × 9 cm was found (Figure 1). At physical examination, heart rate was 82 per minute, blood pressure 150/90 with respiratory rate of 16 per minute, and temperature of 36.3°C. Hirutisim was evaluated based on Ferriman-Gallwey modified score (result = 24). Clitorimegaly, nondeveloped mammary glands, voice deepening and primary amenorrhea were also present (Figures 2, 3, and 4). She had gone through puberty mentally and socially as a male. FSH, LH, prolactin, estradiol, 17-hydroxyprogesterone, and progesterone were normal. Total testosterone was 4.33 ng/mL (0.06–0.82), androstenedione 10 ng/mL (0.4–2.7), and dehydroepiandrosterone sulfate (DHEA-S) > 1000 *μ*g/dL (35–430 *μ*g/dL). Adrenocorticotropic hormone (ACTH), urinary free cortisol, 1 mg dexamethasone suppression test, and 11-deoxycorticosterone were normal. Following a complete removal of telmisartan and metoprolol for 6 weeks, plasmatic methanephrines, urinary metanephrines, and plasma aldosterone concentration/plasma renin activity were found to be normal (Table 1). As well, Doppler ultrasound of renal arteries was found to be without any phytology. After controlling the blood pressure, the patient underwent a successful left adrenalectomy. The specimen was a 10 × 11 cm tumor. Pathology reported an adrenal adenoma with no signs of malignancy. After surgical intervention, plasma DHEA-S and testosterone concentration became normal. At 2-month followup, she was off antihypertensive agents and normotensive and referred that hair growth had clearly stopped.

## 3. Discussion

This case illustrates a long delay in the diagnosis of a virilizing syndrome due to a pure androgen-secreting adrenal adenoma that presented with simultaneous resistant hypertension. Pure androgen-secreting adrenal tumors are extremely rare. Hyperandrogenemia in women may be from an ovarian or adrenal source. DHEA-S, DHEA, androstenedione, testosterone, and dihydrotestosterone are the major circulating androgens in women. DHEA-S, is produced solely in the adrenal gland. DHEA is produced 50% in the adrenal gland, 30% from conversion of DHEA-S and 20% in the ovary. Both glands equally produce androstenedione, and testosterone is synthetized in the adrenal gland (25%), in the ovaries (25%), and 50% from androstendione conversion. Dihydrotestosterone is classically an intracellular androgen [11]. In this case, DHEA-S along with testosterone was elevated. Androgen secreting tumors can produce hirsutism and virilization in 90–100% of the patients and amenorrhea in 40–60% [4, 6]. Our patient had all three of them.

In one of the largest series, Moreno et al., over a period of 33 years, described 21 cases with pure androgen-secreting adrenal tumors. In this paper, they reported 801 adrenalectomies. Only 2.4% were due to pure androgen-secreting adrenal tumors and 4.4% to primary adrenocortical tumors. Almost 50% were malignant, with a mean age of 34.5 ± 17 years in benign tumors, compared to 45 ± 17 in malignant adrenal tumors. Hirsutism was found in all patients, and virilization was only found in 23% of the cases. Tumor size had a mean of 9 cm in the adenomas and 14 cm in the carcinomas. Testosterone and DHEA-S were elevated in virtually every patient. Hypertension was not reported in any case [12]. Cordera et al. reported 11 cases of pure androgen-secreting adrenal tumors over a period of 50 years. In this series, a pure androgen-secreting adrenal adenoma was found for every 318 primary adrenal benign disease adrenalectomies and one for every 12 adrenal carcinomas. Benign tumors were all ≤6 cm, and malignant tumors were ≥8 cm. Hypertension was found in 30% of the cases. Testosterone levels were elevated in 6 cases, and DHEA-S was elevated in 5 patients [6]. Sandrini et al. reported a large series of children with adrenocortical tumors. Mean age was 4.3 years, and virilization was reported in 40% of the cases; most of the tumors were malignant and nonpure secreting androgen tumors [7]. Wajchenberg et al., in a series of adrenocortical carcinoma, found a pure virilization syndrome in 30% of the children and just in four adult patients. Three of these four patients had hypertension. All carcinomas had a size ≥5 cm on its greatest dimension [13]. 

Malignancy likelihood in adrenal tumors has been usually correlated with tumor size. In this sense, adrenal masses greater than 4 cm have been typically associated with malignancy. Even though it has been described that benign pure androgen-secreting adrenal adenomas could have a size close to 10 cm, most of them are smaller [6, 12]. Besides, mixed hormonal secretion is suggestive of malignancy while pure secretion is related to benign adrenal disease. Sciarra et al. reported that adrenal carcinomas mostly presented with elevation of DHEA-S instead of testosterone, while others have not found this feature [13, 14]. In our case, tumor size and the mixed secretion of DHEA-S and testosterone increased the possibility of malignancy, but pure androgen secretion and the indolent and slow progression of the disease over a period of ten years strongly suggested a benign tumor. 

The association between pure androgen-secreting adrenal adenomas and hypertension is extremely rare. This association has just been reported in a few cases and it has usually been related to malignant tumors with a mixed secretion of androgens, glucocorticoids, and/or aldosterone [6, 13]. Compression of the renal artery with concomitant secondary aldosteronisim has been hypothesized as a potential cause in large size tumors [8]. The association and the mechanism of hypertension with pure androgen-secreting adrenal tumors remain unclear [6, 12, 13, 15]. Experimental studies show that prenatal androgen exposure is related in adult life to a moderate increase in blood pressure [16–19]. Similarly, studies have shown that prenatal exposure to androgens is related to a reduced endothelium-dependent vascular relaxation due to a dysfunction in nitric oxide synthase [20]. It is important to mention that it is well known that blood pressure is higher in men than women, but the exact mechanism remains unsolved. A possible association of male sex hormones with reduction of pressure natriuresis as a contributing cause of hypertension has already been proposed [9, 10]. In our patient, aldosterone, cortisol, or catecholamine hypersecretion were clearly ruled out. Therefore, hyperandrogenemia was the most likely etiology for hypertension, although it is difficult to point it out as the only responsible cause of the severe hypertension. It is likely that another nonmeasured precursor metabolite, produced by the tumor, with an effect on blood pressure, could be responsible for severe hypertension since blood pressure normalization rapidly occurred after the removal of this benign adrenal neoplasm.

## 4. Conclusion

 Pure androgen-secreting adrenal tumors are extremely rare. Hirustism, virilization, and menstrual irregularities are the usual clinical findings. Malignancy is difficult to predict, but it is usually related with tumor size, type of hormones secreted, and the velocity of the tumor progression. The association with hypertension is extraordinarily rare, and it is usually accompanied by glucocorticoid or aldosterone hypersecretion. The exact mechanism by which a pure androgen-secreting adrenal tumor can cause hypertension remains unclear.

## Figures and Tables

**Table 1:** Laboratory measures.

Value	Basal	After surgery	Range
FSH (UI/L)	4.41	—	(2–12)
LH (UI/L)	4.24	—	(1–18)
Estradiol (pg/mL)	197.90	—	(15–350)
Prolactin (ng/mL)	21.18	—	(4–30)
17-Hydroxyprogesterone (ng/dL)	25	—	(20–300)
Androstenedione (ng/mL)	10	0.3	(0.4–2.7)
Testosterone (ng/mL)	4.33	0.24	(0.06–0.82)
DHEA-S (*μ*/dL)	>1000	31.3	(35–430)
11-DOC (ng/dL)	5.3	—	(1.5–37)
ACTH (pg/mL)	17.14	—	(10–60)
Progesterone (ng/mL)	1	—	(2–20)
UFC (ug/day)	18.4	—	(36–137)
PM (pg/mL)	21	—	(≤57)
TUM ug/24 hrs	194	—	(25–300)
PAC (ng/dL)	25	—	(2–18)
PRA (ng/mL/hr)	10.5	—	(0.25–5.82)
PAC/PAR	2.38	—	<30

FSH: follicle-stimulating hormone; LH: luteinizing hormone; DHEA-S: dehydroepiandrosterone sulfate; 11-DOC: 11-deoxycorticosterone; ACTH: adrenocorticotropic hormone; UFC: urinary free cortisol; PM: plasma metanephrines; TUM: total urinary metanephrines; PAC: plasmatic aldosterone concentration; PRA: plasmatic renin activity.

## References

[1] Melmed S, Polonsky KS, Reed P, Kronengerg EH *Williams Textbook of Endocrinology*.

[2] Young WF (2007). Clinical practice. The incidentally discovered adrenal mass. *The New England Journal of Medicine*.

[3] Barzon L, Sonino N, Fallo F, Palù G, Boscaro M (2003). Prevalence and natural history of adrenal incidentalomas. *European Journal of Endocrinology*.

[4] Derksen J, Nagesser SK, Meinders AE, Haak HR, Van De Velde CJH (1994). Identification of virilizing adrenal tumors in hirsute women. *The New England Journal of Medicine*.

[5] Calhoun DA, Jones D, Textor S (2008). Resistant hypertension: siagnosis, evaluation, and treatment a scientific statement from the american heart association professional education committee of the council for high blood pressure research. *Hypertension*.

[6] Cordera F, Grant C, van Heerden J (2003). Androgen-secreting adrenal tumors. *Surgery*.

[7] Sandrini R, Ribeiro RC, DeLacerda L (1997). Extensive personal experience: childhood adrenocortical tumors. *Journal of Clinical Endocrinology and Metabolism*.

[8] Hutter AM, Kayhoe DE (1966). Adrenal cortical carcinoma. Clinical features of 138 patients. *The American Journal of Medicine*.

[9] Staessen J, Fagard R, Lijnen P, Thijs L, Van Hoof R, Amery A (1990). Reference values for ambulatory blood pressure: a meta-analysis. *Journal of Hypertension*.

[10] Wnnberg N, Hoegholm A, Chmtensen HR (1995). 24-h ambulatory blood pressure m 352 normal Damsh subjects, related to age and gender. *American Journal of Hypertension*.

[11] Burger HG (2002). Androgen production in women. *Fertility and Sterility*.

[12] Moreno S, Montoya G, Armstrong J (2004). Profile and outcome of pure androgen-secreting adrenal tumors in women: experience of 21 cases. *Surgery*.

[13] Wajchenberg B, Pereira M, Medonca B (2000). Adrenocortical carcinoma: clinical and laboratory observations. *Cancer*.

[14] Sciarra F, Tosti-Croce C, Toscano V (1995). Androgen-secreting adrenal tumors. *Minerva Endocrinologica*.

[15] Zografos GC, Driscoll DL, Karakousis CP, Huben RP (1994). Adrenal adenocarcinoma: a review of 53 cases. *Journal of Surgical Oncology*.

[16] Davies MJ, Marino JL, Willson KJ, March WA, Moore VM (2011). Intergenerational associations of chronic disease and polycystic ovary syndrome. *PLoS ONE*.

[17] Geelhoed JJM, Fraser A, Tilling K (2010). Preeclampsia and gestational hypertension are associated with childhood blood pressure independently of family adiposity measures: the Avon longitudinal study of parents and Children. *Circulation*.

[18] Lawlor DA, Macdonald-Wallis C, Fraser A (2012). Cardiovascular biomarkers and vascular function during childhood in the offspring of mothers with hypertensive disorders of pregnancy: findings from the Avon Longitudinal Study of Parents and Children. *European Heart Journal*.

[19] Mamun AA, Kinarivala MK, O’Callaghan M, Williams G, Najman J, Callaway L (2012). Does hypertensive disorder of pregnancy predict offspring blood pressure at 21 years? Evidence from a birth cohort study. *Journal of Human Hypertension*.

[20] Sathishkumar K, Elkins R, Yallampalli U, Balakrishnan M, Yallampalli C (2011). Fetal programming of adult hypertension in female rat offspring exposed to androgens in utero. *Early Human Development*.

